# Supplementary material for: The Influence of Race/Ethnicity on the Transcriptomic Landscape of Uterine Fibroids
Source: Int J Mol Sci. 2023 Aug 30;24(17):13441. doi: 10.3390/ijms241713441 (PMC10487975; doi:10.3390/ijms241713441)
Supplement: Supplementary file 1 [file ijms-24-13441-s001.zip › Supplementary Figures and legends.pdf]

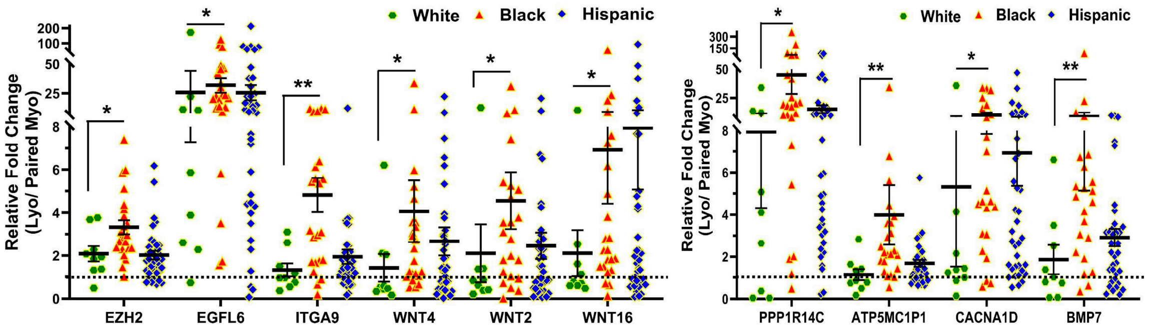

Supplementary Figure S1: The mRNA expression of *EZH2, EGFL6, ITGA9, WNT4, WNT2, WNT16, PPP1R14C, ATP5MC1P1, CACNA1D* and *BMP7* expressed as fold change (Lyo/ paired Myo) in White (n=9), Black (n=23) and Hispanic group (n=37) by qRT-PCR. The results are presented as mean ± SEM with P values (*P<.05; **P<.01; ***P<.001) as indicated by the corresponding lines.

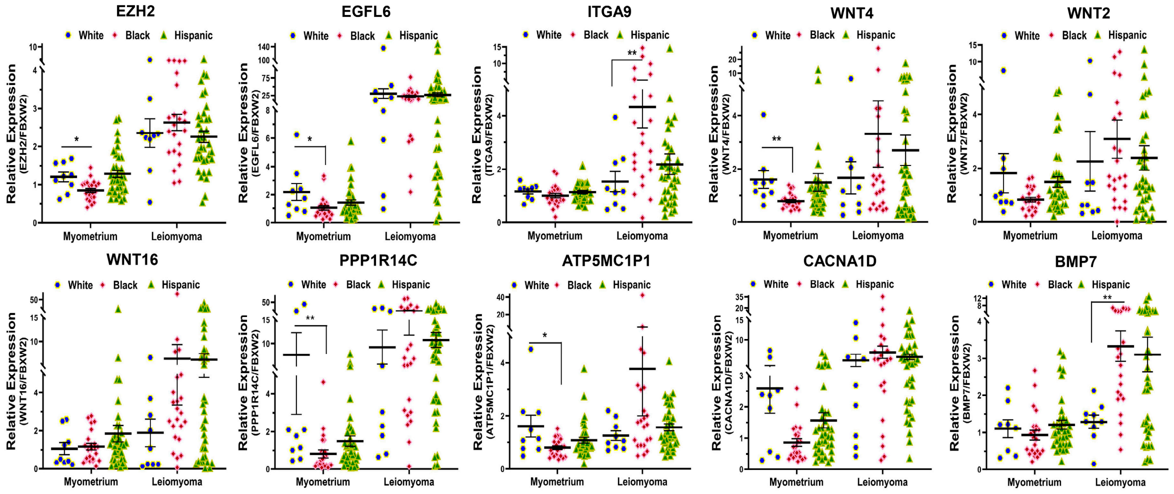

Supplementary Figure S2: The mRNA expression of *EZH2, EGFL6, ITGA9, WNT4, WNT2, WNT16, PPP1R14C, ATP5MC1P1, CACNA1D* and *BMP7* expressed in myometrium and leiomyomas in White (n=9), Black (n=23) and Hispanic group (n=37). The results are presented as mean ± SEM with P values (*P<.05; **P<.01) as indicated by the corresponding lines.

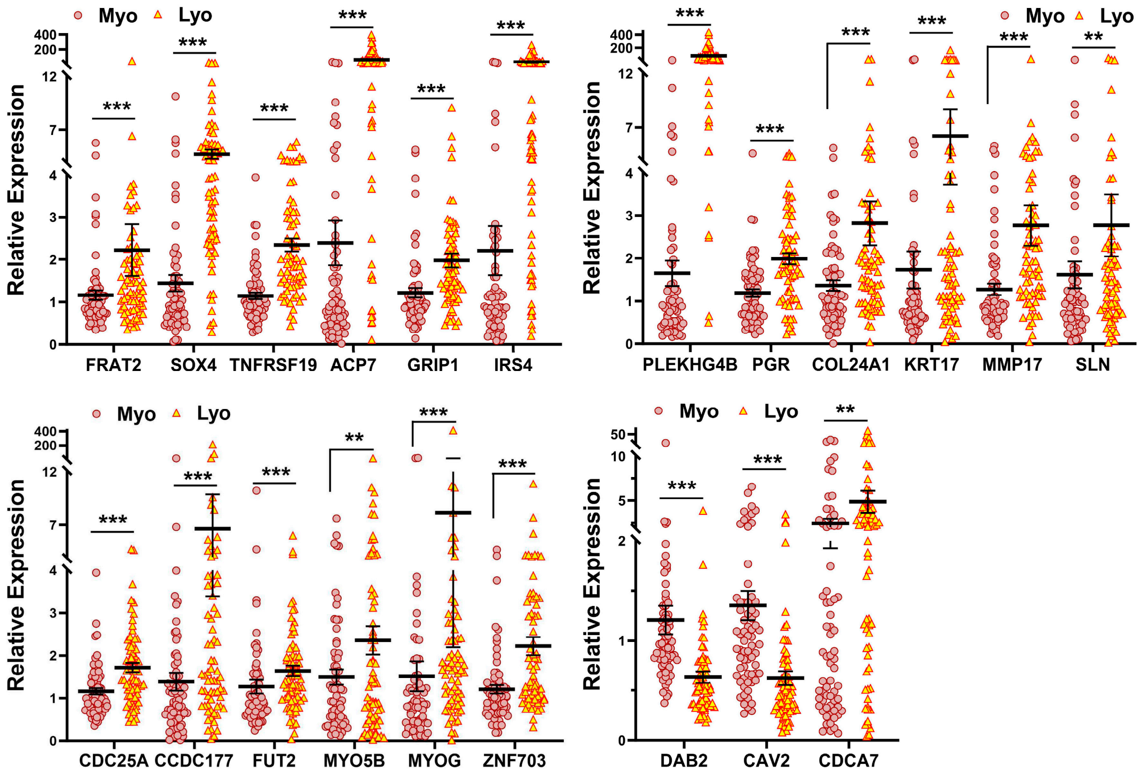

Supplementary Figure S3: The expression of select genes (*FRAT2, SOX4, TNFRSF19, ACP7, GRIP1, IRS4, PLEKHG4B, PGR, COL24A1, KRT17, MMP17, SLN, CDC25A, CCDC177, FUT2, MYO5B, MYOG, ZNF703, DAB2, CAV2* and *CDCA7*) comparing myometrium with its paired leiomyomas (n=69) by qRT-PCR. The results are presented as mean ± SEM with P values (**P<.01; ***P<.001) indicated by corresponding lines.

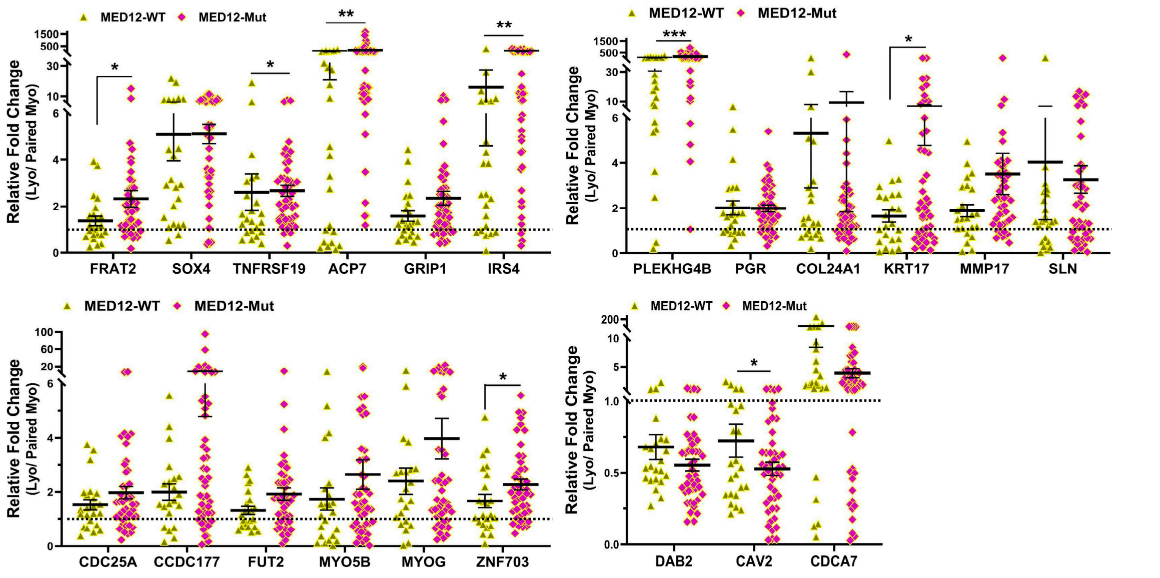

Supplementary Figure S4: The expression of mRNA for *FRAT2, SOX4, TNFRSF19, ACP7, GRIP1 and IRS4, PLEKHG4B, PGR, COL24A1, KRT17, MMP17 and SLN, CDC25A, CCDC177, FUT2, MYO5B, MYOG and ZNF703, DAB2, CAV2* and *CDCA7* expressed as fold change (Lyo/ paired Myo) in MED12 mutated (n=46) and non-mutated (n=23) specimens by qRT-PCR. The results are presented as mean ± SEM with P values (*P<.05; **P<.01; ***P<.001) as indicated by the corresponding lines.
